# Supplementary material for: Risk factors for sacrococcygeal pilonidal sinus: a systematic review and meta-analysis supplemented by genetic causal assessment
Source: Front Surg. 2026 Jan 7;12:1718589. doi: 10.3389/fsurg.2025.1718589 (PMC12819706; doi:10.3389/fsurg.2025.1718589)
Supplement: Supplementary file 2 [file Datasheet2.zip › Supplementary Data 2/MR_pipeline_p5e-8/forest_plots/ebi-a-GCST006097.finngen_R12_L12_PILONIDALCYST.pdf]

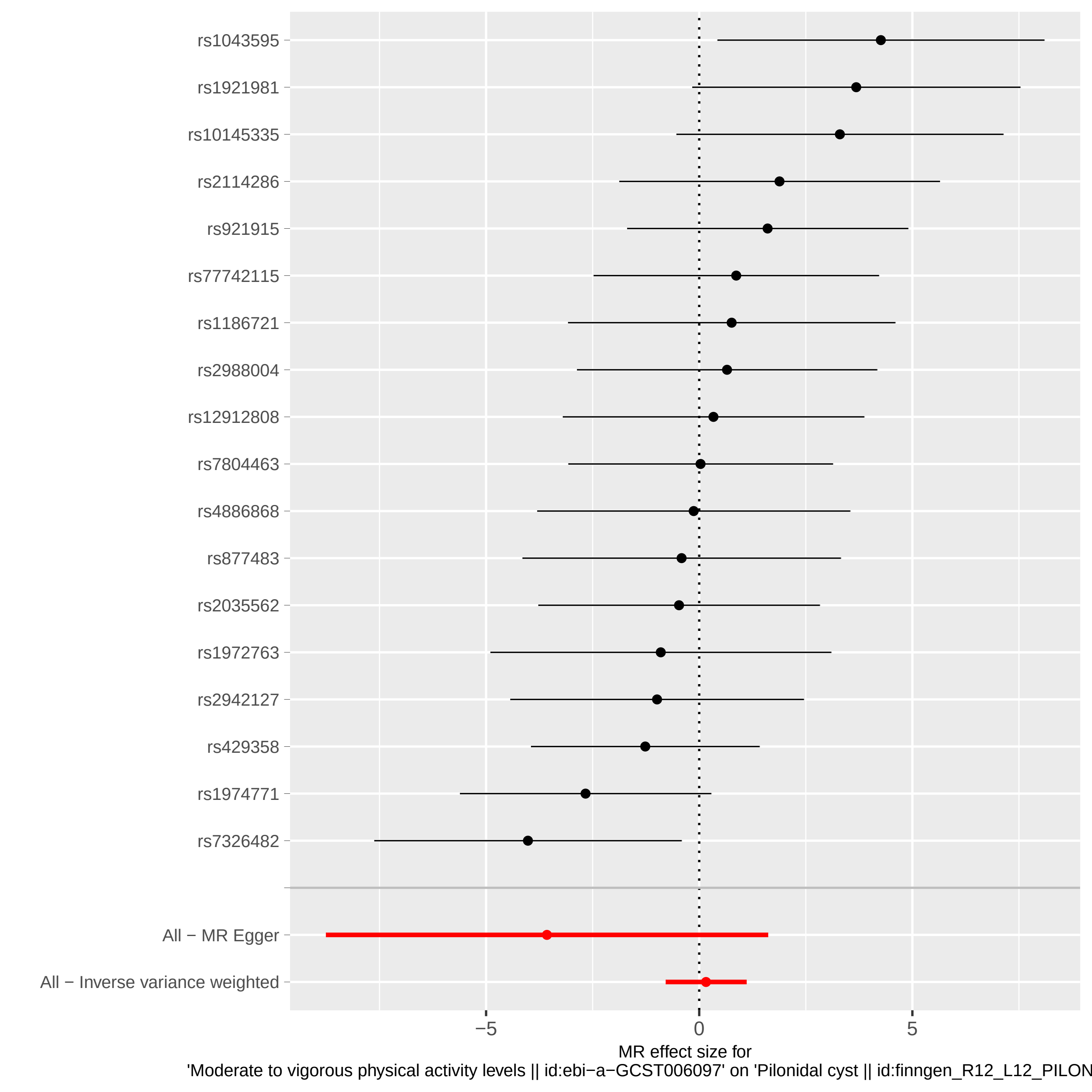
rs1043595
rs1921981
rs10145335
rs2114286
rs921915
rs77742115
rs1186721
rs2988004
rs12912808
rs7804463
rs4886868
rs877483
rs2035562
rs1972763
rs2942127
rs429358
rs1974771
rs7326482
All − MR Egger
All − Inverse variance weighted
−5
0
5
MR effect size for
'Moderate to vigorous physical activity levels || id:ebi−a−GCST006097' on 'Pilonidal cyst || id:finngen_R12_L12_PILON
